# The data management of a phase III efficacy trial of an 11-valent pneumococcal conjugate vaccine and related satellite studies conducted in the Philippines

**DOI:** 10.1186/1756-0500-5-274

**Published:** 2012-06-07

**Authors:** Diozele Hazel M Sanvictores, Marilla G Lucero, Hanna Nohynek, Veronica L Tallo, Antti Tanskanen, Leilani T Nillos, Gail Williams

**Affiliations:** 1 Research Institute for Tropical Medicine, Muntinlupa City, Philippines; 2 National Institute for Health and Welfare, Helsinki, Finland; 3 Karolinska Institutet, Stockholm, Sweden; 4 University of Queensland, Brisbane, Australia

## Abstract

**Background:**

A large phase III placebo-controlled, randomized efficacy trial of an investigational 11-valent pneumococcal conjugate vaccine against pneumonia in children less than 2 years of age was conducted in the Philippines from July 2000 to December 2004. Clinical data from 12,194 children who were given either study vaccine or placebo was collected from birth up to two years of age for the occurrence of radiologically proven pneumonia as the primary endpoint, and for clinical pneumonia and invasive pneumococcal disease as the secondary endpoints. Several tertiary endpoints were also explored. Along the core trial, several satellite studies on herd immunity, cost-effectiveness of the study vaccine, acute otitis media, and wheezing were conducted.

**Results:**

We describe here in detail how the relevant clinical records were managed and how quality control procedures were implemented to ensure that valid data were obtained respectively for the core trial and for the satellite studies. We discuss how the task was achieved, what the challenges were and what might have been done differently.

**Conclusions:**

There were several factors that made the task of data management doable and efficient. First, a pre-trial data management system was available. Secondly, local committed statisticians, programmers and support staff were available and partly familiar to clinical trials. Thirdly, the personnel had undergone training during trial and grew with the task they were supposed to do. Thus the knowledge needed to develop and operate clinical data system was fully transferred to local staff.

**Trial registration:**

Current Controlled Trials ISRCTN62323832

## Background

*Streptococcus pneumoniae* causes high morbidity and mortality from pneumonia and invasive disease among young children particularly in low income countries [1]. Preventive measures, such as vaccination, are important strategies for the control of pneumococcal disease [2]. A phase III clinical trial was conducted in the Philippines to evaluate the efficacy of an investigational 11-valent pneumococcal conjugate vaccine (11PCV) against pneumonia in children less than two years of age [3]. The sponsor of this trial was the ARIVAC (Acute Respiratory Infection Vaccine) consortium, which consists of five international collaborators: the Research Institute for Tropical Medicine (RITM), Philippines; the National Institute for Health and Welfare (THL, formerly KTL), Finland; the University of Queensland (UQ), Australia; the University of Colorado (CU), USA, and the developer and manufacturer of the 11PCV, Sanofi Pasteur, France.

During the trial, various operational challenges were encountered by the investigators. These included the trial infrastructure preparation and implementation according to Good Clinical Practice requirements, communication in the context of a multinational collaboration over wide time zone distances, needs of training and supervision, as well as the logistic constraints typical of a low income country. The trial protocol had one primary endpoint, which drove the sample size, as well as several exploratory secondary, and tertiary objectives. In addition, several satellite studies were nested in or conducted alongside the core trial. These included a herd immunity study, a nested immunogenicity and reactogenicity study, an epidemiologic disease burden study of pneumonia, suspected meningitis and sepsis, a cost-effectiveness study, and the studies on Acute Otitis Media (AOM), wheezing and pertussis. All this taken together posed a considerable challenge to the way data were managed in the trial.

The primary task of data management was to assure that clinical endpoints detected at the hospital emergency ward or during outpatient consultations were events that were coming from children enrolled in the trial (i.e. from the genuine trial subjects), and that the vaccination status was determined correctly. Since the trial included various records of the trial subjects (such as baseline infancy data sheets, health record information, vaccination records, etc.), issues of quality and reliability of the data were also important aspects that had to be monitored. Moreover, since the project was not only concerned with trial data but other studies as well, linkage of records across studies and implementation of quality controls were also crucial to follow-up. The aim of this article is to document the processes and lessons learned from the data management of the various studies conducted in the Philippines under the ARIVAC consortium.

## Results

### Study setting of the core trial

**Figure 1:**
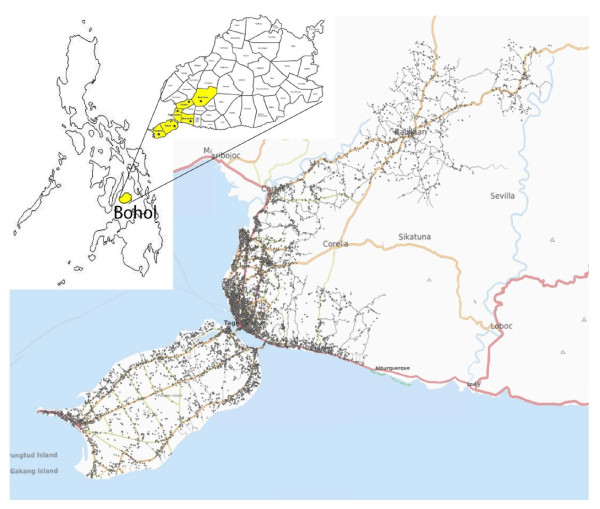
**The map of the trial area.** The trial participants’s homes are marked with black dots. The geographic data was collected later, during the years 2008-2009. In the insert, map of the Philippines showing Bohol and in the Bohol map the 6 municipalities included in the trial are colored with yellow. Base map: http://www.openstreetmap.org/?lat=9.6708&lon=123.9147&zoom=12&layers=M.

The core trial conducted on the Island of Bohol, central Philippines (Figure 1) was a phase III individual randomized double-blind, placebo controlled trial (RCT) of an 11PCV against pneumonia in children less than 2 years of age (ISRCTN62323832). Enrolment of the trial subjects was conducted from July 2000 to December 2003. They were followed up until December 2004. The detailed conduct and the main results of the trial have been described previously by Lucero et al. [3]. In brief, vaccination was administered in 48 barangay health stations (BHSs) in 6 municipalities in the province. The primary endpoint in the core trial was radiologically proven pneumonia, diagnosed in one tertiary care 250-bed government hospital [i.e. admission and outpatient departments (OPD) of the Bohol Regional Hospital (BRH)], and in three smaller 50-bed private hospitals.

### Study population

In the trial cohort, children were divided into three major study groups. The primary study group [i.e. the RCT group] comprised of those who were recruited and enrolled in the efficacy trial of the 11PCV against pneumonia (N = 12,194). A subset of the RCT group of 1,111 children was enrolled into the nested immunogenicity study according to their place of residence (three areas were chosen for this; for further details see Ugpo et al. [4]). The second study group [named EPI group] comprised of children who either refused or were excluded from enrolment in the RCT (N = 577). This group were collected routine vaccinations they availed from the immunization program of the government. Their hospitalization and consultation in the hospitals under endpoint surveillance were also collected. The third study group [named the EPI grey study group] comprised of children who had either pneumonia, suspected meningitis, suspected sepsis, AOM or pertussis, and who came for hospitalization or consultation in the hospitals under endpoint surveillance but were not enrolled in the RCT at the time of the event. These children had either not been enrolled in the RCT at the time of the hospitalization or consultation or were too old to qualify. The children in the third study group did not have vaccination records in the database and only minimum demographic information was collected at every contact with the child (such as the name of the child, date of birth, sex, address, name of mother and if he/she received first Diphtheria-Tetanus-Pertussis (DTP) vaccination). Each hospitalization or consultation was considered to be independent and no attempt was made to inter-link the records.

### Satellite studies

Several additional satellite studies were conducted in conjunction with the core trial (Table 1).

**Table 1:** Description of the study populations

**Study**	**Study areas**	**Age of population**	**Study group**
Phase 3 RCT study			
Efficacy study	6 study municipalities	Children aged 6 weeks to 6 months and monitored until 2 years old	RCT
Nested study	3 predefined health centres included Dao (Tagbilaran), Danao and main health centre in Panglao	Children aged 6 weeks to 6 months and monitored until 2 years old	RCT
Hospitalization in BRH	6 study municipalities	Children less than 5 years old	RCT, EPI and Epi grey
Hospitalization in the private hospital and OPD consultation in BRH	6 study municipalities	Children less than 2 years old	RCT, EPI and Epi grey
Satellite studies			
Herd immunity	6 study municipalities	Children enrolled in the efficacy study w/elder sibling enrolled in the efficacy study	RCT
Cost effectiveness(hospitalization for pneumonia, sepsis, or meningitis at BRH and three private hospitals)	6 study municipalities	Children enrolled in the efficacy study from 6 weeks to 23 months old and admitted to hospital or consulted the OPD	RCT
Acute otitis media(hospitalization or consultation at BRH for pneumonia, sepsis, or meningitis)	6 study municipalities	Children less than 5 years old diagnosed with pneumonia and/or AOM	RCT, EPI and Epi grey
Wheezing(hospitalization or consultation at BRH for pneumonia, sepsis, or meningitis)	6 study municipalities	Children less than 5 years old	RCT, EPI and Epi grey
Pertussis(hospitalization or consultation at BRH for pneumonia, sepsis, or meningitis)	6 study municipalities	Children less than 13 years old	RCT, EPI and Epi grey, children not necessary enrolled in RCT

#### Herd immunity

Starting in January 2002, the field nurses recruited subjects for the herd immunity study. There were 1,843 infants enrolled in the efficacy trial with elder sibling who enrolled in the herd immunity study. The herd immunity study was conducted to assess the possible herd immunity effects of the 11PCV (i.e. reduction of vaccine type/group-specific carriage of *Streptococcus pneumoniae* in the population; http://www.pneumocarr.org). This study assessed the effect on the siblings of the nasopharyngeal pneumococcal carriage of a younger sibling who had been vaccinated with the 11PCV. Nasopharyngeal swabs for detection of pneumococcal carriage were collected at the enrolment (6 weeks of age) and at the measles vaccination visits (9 months of age).

#### Cost effectiveness

The cost effectiveness study started in October 2001 and details of hospitalization and consultation of those enrolled in RCT group were collected until September 2004. There were a total of 1,499 hospitalizations and consultations for pneumonia, suspected meningitis and/or suspected sepsis that were included in the cost effectiveness study. The data collected were the length of hospitalization, diagnostic tests, special services and procedures done, physician’s fee, pharmaceutical costs, oxygen use and devices used during hospitalization and consultation as well as parental loss of income.

#### Miscellaneous studies

Children in the RCT, EPI and EPI grey study groups who remained in the trial area, were enrolled in a few other studies (i.e. AOM, wheezing and pertussis studies, respectively) until they reached the age of 5 years.

The AOM and wheezing studies started in February 2002 and children enrolled were diagnosed with pneumonia and/or AOM. The study subjects were less than 5 years old. The pertussis study started in July 2001 with a study population that included children less than 13 years old. This study included older children (not necessary enrolled in the RCT) who qualified the criteria for pertussis. Data for AOM, wheezing and pertussis were collected until December 2004. In the AOM and wheezing studies, 2,018 children with one or more than episodes of hospitalizations and consultations were enrolled. The AOM and wheezing studies aimed at determining the presence of AOM and wheezing in children less than 5 years who were hospitalized at BRH or consulted at BRH-OPD. The data collected included presence of cough, duration of cough, ear pain, irritability, vomiting, methods of ear examination, diagnosis of ear examination, laboratory samples collected and episodes of wheezing. In the pertussis study, 219 children with one or more than episodes of hospitalizations and consultations were enrolled. The data collected included duration of cough, presence of paroxysmal cough, vomiting (induced by cough), whooping cough, and suspicion of pertussis in the patient and/or among the household members.

### Operational processes

#### Recruitment and enrolment

All births occurring since February 2000 in the six study municipalities were followed up for possible recruitment in the core trial. Basic demographic information and the intention of the parents/guardians to have their child participate in the trial were collected. Information on the place of residence and the first DTP vaccination were obtained from the parents/guardians. This was usually done during home visits by the study nurse when she talked with the parents/guardians about the trial. These activities were undertaken by the study nurses to facilitate recruitment and to give time to the parents/guardians to make decision before enrolment.

During the first vaccination visit, the study nurse or study physician ascertained that the infant fulfilled all the inclusion criteria, did not present with the exclusion criteria, and that the parent/guardian signed the informed consent form. The study nurse filled in an infant data information sheet containing basic demographic data of the child and the mother, and assigned unique child identification (Child_ID) number. Based on the randomization list, which was produced by the pharmaceutical partner of the trial, the nurse allocated the child to either receive the study vaccine (11PCV) or the saline placebo. The infant data information sheet constituted the primary record of the child. During the first visit, the study nurse also issued to the parent/guardian a health card (commonly known as the “yellow card”) with a Child_ID sticker pasted on the card. The vaccinations received, among other information, were recorded into this card. To identify the child’s enrolment in the trial, the parent/guardian was requested to always bring the child’s yellow card with them whenever the child visited the health centre or hospital.

#### Vaccination

After enrolment, the child received the first dose of the trial vaccine or placebo at the minimum of 6 weeks of age. The second dose was scheduled to be given 4 weeks after the first dose and the third dose 4 weeks after the second dose. Administration of the study vaccine and concomitant vaccines (BCG, OPV, DTP/PRP ~ T, HBV, measles and vitamin A) were recorded on the vaccination case report form (CRF).

Those not enrolled in the trial received routine Expanded Program of Immunization (EPI) vaccines (BCG, OPV, DTP, HBV, measles and vitamin A) available from the health centre supply. This data was collected and recorded on a shorter version of the vaccination CRF.

Before vaccination, the child was assessed for the presence of any permanent or temporary contraindications. The study nurse had a list of questions to assist her in assessing the eligibility of the child to receive the vaccination. The presence or absence of contraindications was recorded on the contraindication CRF. If there was a temporary contraindication, the vaccination was postponed to the next scheduled health centre vaccination visit (~4 weeks later). If there was an absolute contraindication, the child was excluded from the trial.

#### Hospitalization and consultation due to pneumonia

Hospitalizations due to pneumonia at BRH and the 3 private hospitals were monitored daily for children less than 5 years. Consultations in the out-patient department of the BRH (BRH-OPD) were monitored only on working days. Each hospitalization and consultation of the child was given a unique identification (chronological order) number. A child could have several hospitalizations during the trial. Hospitalization from BRH was recorded using a different CRF, because besides pneumonia, also initial diagnoses of meningitis and sepsis were recorded. For hospitalizations in the private hospitals and the BRH-OPD only pneumonia cases were recorded.

Any hospitalization in the BRH due to pneumonia in children not enrolled in the core trial and aged 6 weeks to 59 months was collected for the epidemiology study. Also consultations of pneumonia in the BRH-OPD and hospitalizations due to pneumonia in the 3 private hospitals for children aged 6 weeks to 23 months were included in the epidemiology study. Initial diagnoses of meningitis and sepsis in the BRH were also included in the epidemiology study.

#### Laboratory sample collection during hospitalization and consultation

Several samples [blood, pleural fluid, nasopharyngeal swabs and aspirate, urine and cerebrospinal fluid (CSF)] for laboratory tests were collected during hospitalization due to pneumonia, meningitis and/or sepsis in the BRH. For OPD consultations nasopharyngeal aspirate was collected using systematic sampling of every 5^th^ patient. These were recorded using a laboratory CRF and indexed with the same hospitalization identification number compared to the hospitalization or consultation.

#### Serious adverse events (SAEs)

An SAE was defined as any untoward medical occurrence after immunization that resulted in death, was life threatening, required in-patient hospitalization or prolonged existing hospitalization, or resulted in persistent or significant disability/incapacity. SAEs were monitored in eight hospitals in the study area as well as in the 48 barangay health stations. Information on hospitalizations of study infants from 6 weeks to two years of age belonging to the RCT group was identified, followed up and recorded by using a screening log form. During vaccination days, study nurses questioned barangay health stations’ staff about any SAEs and deaths in the area. SAEs were reported to the safety monitoring team of local and Finnish physicians who will review and classify the SAE for periodic or immediate reporting to the vaccine manufacturer, Sanofi Pasteur. Periodically reported SAEs are reported monthly while immediately reported SAEs are reported within 48 h from the time the event become known to the investigators.

### Clinical data management system (CDMS)

#### Description of the CDMS

Data collection and information system requirements originated from the need of regular, effective and timely monitoring and reporting during the trial. This was also essential for the statistical analyses once the trial was completed. The primary goal of the clinical data management system (CDMS) was to allow these reporting requirements to be met, while ensuring the integrity and validity of the data being collected. It was necessary that multiple users were simultaneously able to make modifications to the data and to generate reports from real time data in the database [5]. A decision was made to recommend a specific data management system instead of purchasing a commercial CDMS [6].

The scope of the CDMS was defined by the “data dictionaries” (DDs; in total 57 for all studies) containing metadata of CRFs and by other general reporting or informational requirements. The DDs contained description of forms and other information sources of each study and contained short description of the data: variable names, short explanation, code lists, legal values, format and length. The DDs were maintained by the data manager (DMG) and were defined in conjunction with the trial investigators. The DDs were created alongside the paper forms, such as CRFs. The CRFs were used to initially collect the data by trained nurses, technicians and doctors before entry into the CDMS. To facilitate entry, the data entry screens were one-to-one matches for the paper forms (i.e. the data entry screen looked similar to the paper format). The database structure, tables and definitions of fields were developed from DDs. The database was not normalized (i.e. more like wide form in statistics datasets) [7] and the tables were almost one-to-one with paper forms. The number of tables in the database was over 60 including code lists and the total number of fields in the database reached 2,300.

A provisional database had to be implemented in the start of the core trial, because the data management system contracted from a third party (systems developer in Australia) was not yet complete and in order to report in time the progress of the trial. The provisional database was used only for 2 months and the final version of the Microsoft SQL server database was installed in Bohol Data Centre (BDC) in October 2000. Data entered first in the provisional database was re-entered in the final database due to the differences in the system and definitions.

Reporting was accomplished by exporting the relevant data tables from the Microsoft SQL Server database to a Microsoft Access database, which were then transformed and used by SAS scripts to generate reports in HTML format. The reports were generated monthly and delivered to the relevant members of the trial consortium for monitoring, informational purposes and other requirements.

#### System overview

The Relational Database Management System (RDMS) consisted of Microsoft SQL Server 7 and a Visual Basic 6 front end combined with various Microsoft Access 2000 client fields. The operating system was Windows NT Server 4.0 for the database server and Windows NT 4 Workstation for the clients.

There was also an application for checking the edits, which was created using Microsoft Access 2000. This application contained miscellaneous reporting tools, such as the “Vaccination Schedules” generator. Most importantly, it contained a module for double entry validation that was for checking of discrepancies between the first and second entry data.

To keep track of changes in the database and to restrict privileges, each user had an account and changes were audited with the track including the following information: the site where the data was created, the date when the record was created, the person who modified the record and the date of modification.

#### Personnel

The BDC was composed of a senior data manager, assistant data managers, system administrator, and data entry persons. In general, the staff performed often more than just a one major function. For example the data managers also performed SAS programming and data analyses. This was deemed advantageous because knowledge of the different studies and in-depth understanding of contents, history, restrictions and the relationships of the data were necessary to perform these functions. The trial had a high retention of its personnel due to good working conditions and training provided. Hiring of additional personnel was necessary during the peak period of the data entry.

The personnel were continuously trained to use the CDMS, to handle data management tasks, to do SAS programming and to administrate the database system. Close monitoring of the UQ counterpart was necessary to maintain the high performance of the data management unit.

#### Supervision

The BDC was under the direct supervision of the UQ and the RITM. The BDC data manager liaised with the international and local consultants. Quarterly visits in the BDC were conducted by the UQ personnel to monitor the activities, to directly train personnel and eventually to transfer the responsibility of managing the data and maintaining the database system. The RITM investigators conducted monthly visits in the BDC to monitor the activities and to resolve any administrative issues. In the middle of 2003, most of the trial data management was handled by the BDC. The UQ remained as a consultant and was regularly updated on the activities at the study site.

### Quality control

#### Management of CRF

The CRFs were in triplicate paper copies, which served as the backup copies for the consortium members (Table 2). After the CRFs were filled, the supervisor reviewed the forms before they were submitted for the data entry at the BDC. For the practical reasons and clarity, the first copy was used as a reference for the data entry. After the data entry, the first copy was stored in a different building to ensure that backup copy was safe.

**Table 2:** Case report form (CRF) types submitted per child

**Event**	**CRF**
First vaccination visit	Birth event report
	Infant data sheet
	Vaccination CRF
	Herd immunity (if applicable)
Second vaccination visit	Vaccination CRF
Third vaccination visit	Vaccination CRF
Measles vaccination visit	Measles CRF
	Herd immunity (if applicable)
Postponement visit	Postponement CRF
Hospitalization in BRH	Hospitalization CRF
	Radiology
	Laboratory
	Bacteriology
	Cost effectiveness
	AOMW (if applicable)
	Pertussis (if applicable)
Hospitalization in private hospitals	Hospitalization CRF
	Radiology
	Cost effectiveness
OPD consultation in BRH	Hospitalization CRF
	Radiology
	Laboratory (if applicable)
	Bacteriology (if applicable)
	Cost effectiveness
	AOMW (if applicable)
	Pertussis (if applicable)
Completion/Termination visit	Completion CRF
Any sae	Serious Adverse Event Report
Death	Death event report
	Verbal autopsy (if applicable)

Submission of vaccination CRFs was done by the centre visits per child rather than separately by forms. For the hospitalizations, submission of CRF was also per hospitalization and included laboratory and bacteriology CRFs, if required. Usually a certain schedule was followed with the submissions of the CRFs to give time for data entry, cleaning and validation before the production of monthly reports.

#### Data entry

All CRFs from the different studies (of the children enrolled in the core trial) were entered twice by two independent data entry officers. For the remaining subjects, any CRFs arising from participation in any of the other studies were single-entered in the database. The birth and death records, which were collected from the public documents (e.g. the church, civil register, hospital and health centre records) were single-entered. The data entry contained integrity checks, range checks and business rules of the trial.

The core data were stored in the database server in two separate databases: the first entry and the second entry database. This process enabled separate entries, since the data entry was not dependent on whether the data were already entered in the primary database (which was the first entry for this study). For the remaining subjects, all data were entered in the primary database only.

**Figure 2:**
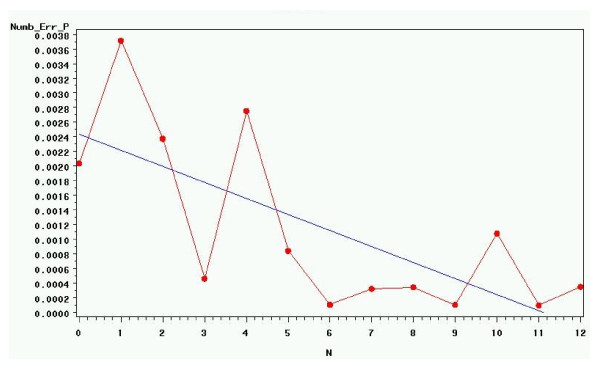
**The monthly error rates in Numeric Fields of the “*Child Data*” in the First 12 Months of Double Data Entry**.

After the first 12 months of double data entry, an assessment was made about the continuation of double data entry in light of the very minor errors. The conclusions of the assessment were that it took six to seven months for error rates to stabilize and that there was considerable variation by the type of CRF. Particularly there were high error rates in the Infant Data Sheet, Serious Adverse Event and Admission CRFs. The results were presented to the Data Safety and Monitoring Board (DSMB) in July 2001 [8]. It was decided by the consortium to retain double data entry as one of quality measures implemented on the data. Double data entry was suggested to ensure and reassure that data on the CRFs were correctly transcribed into electronic format, monthly error rates of child data are shown in Figure 2.

#### Validation and cleaning of the data

**Figure 3:**
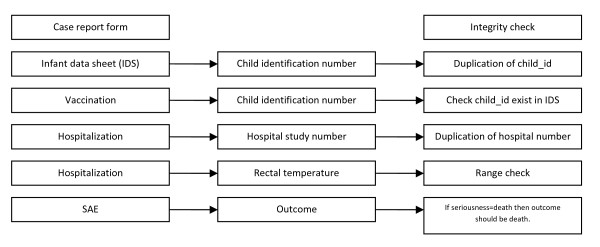
**An example of the integrity checks inherent in the database system**.

The data validation method was done stepwise during entry, periodically in the database and with separate logic in SAS depending on the nature of errors traced [9]. The validation was done on the entire database of both entries from the very beginning of the study to the most current data. The first stage of validation compared the first and second entries for the data that were double-entered. The data was transformed to SAS format for validation and data validation procedures were written in SAS language using the comparison procedure to compare the two entries. Comparison was made variable by variable in each table in the databases and a report was generated indicating the variable and its value that did not match within the databases. Since comparison was done on the entire database, any correction or updates on the data definition were implemented on the entire database. Audit trails were produced to document data entry and correction. Once both entries had been exactly matched, the first entry (which included all data of both enrolled and not enrolled children) was used to perform data analysis. Series of validation procedures were developed and performed in validation of CRFs, range checks, inconsistencies, and univariate and multivariate checks. This was an addition to the CDMS integrity, checks which were concurrently applied during data entry (Figure 3). Validations were run on the entire database monthly. These validations were regularly reviewed and updated by investigators who were experts in a particular CRF or study and by the data managers who had in-depth knowledge of the database.

On-site and monthly validation and cleaning of the database contributed advantageously to the project, because the volume of CRFs to be corrected was manageable for the data managers, and for the field and hospital personnel who collected the data. It was also easier for the field and hospital personnel to recall the recent data or go back to the source, when required. An independent data monitoring team (IDMT) which was formulated on the recommendation of the DSMB randomly checked a certain percentage of case report forms monthly.

#### Correction of the CRF entries

Corrections of the CRF entries were performed directly on the paper CRF following a standard operating procedure (SOP). In order to keep tract of any corrections and movement of the CRF, a query sheet was designed to record this information. The query sheet was attached to the CRF and issued by the data manager. The query sheet was a preformatted form and filled-in with information on the data to be corrected, reason for its error, name of the CRF and name of the study nurse who completed the CRF. The query sheet was given to the personnel who made the correction. The information on the query sheet was stored in the database and it was also used as a tool to evaluate the performance of the study nurses.

There were 4,508 data queries that were issued from December 2000 to February 2005. The most common errors were wrong entries. Other errors were inconsistent entries within the form and/or compared to other forms. Quality assurance on the filling of the BDC copies of the CRFs was performed on a quarterly basis. Ten percent of the forms filed at the BDC office were manually checked by the data managers for completeness and consistency.

#### Reporting

Monthly trial progress summary and monthly total numbers of birth, recruitment, vaccination, SAE, pneumonia admission and consultation, completion and termination were reported. Other reports produced were quarterly summaries of the data safety monitoring board (DSMB) and operational reports. At the start of the trial, the reports were sent out to the consortium via email in a secure encrypted form. Later on, when the ARIVAC website was constructed, the reports were posted in the secure area.

#### Code breaking

When analysing the data, sessions of code breaking are organized to maintain and keep the trial vaccine/placebo codes blinded. The goal of blinded analysis is to ensure that any person involved in developing analyses is not at any stage exposed to the true values of the trial vaccine/placebo codes. The 11PCV/placebo was randomized using letter codes from A to F (3 letters for 11PCV and placebo). The vaccines bearing these letter codes were allocated to the children according to a scratchable randomization list provided by Sanofi Pasteur.

Analysis scripts were developed using the scrambled version of the Master database, where the key variable of 11PCV/placebo was replaced with randomly allocated letter. After the analysis scripts were tested, the scripts were run over the complete data with real 11PCV/placebo codes. A third person external to the project was hired to make this codebreak and ensure that the data remained blinded.

This person opened and used the 11PCV/placebo codes to run the analysis, and ensured that the 11PCV/placebo codes were secured after the analysis was complete. Outputs from the unblinded analysis were distributed to the ARIVAC consortium. To protect the integrity of the primary objective of the trial, the manuscript on the primary results was first published before unblinded data were given to researchers.

## Discussion

Clinical data management of this 11PCV efficacy trial and related satellite studies was challenging, because of the magnitude of data collection and the corresponding complexities presented by the simultaneously conducted studies. One of the challenging tasks was to keep track of children with multiple clinical events, i.e. all the different outcomes studied. These multiple clinical events and all information from the different studies had to be linked accurately. It was laborious to maintain the quality of the data, and the linkage of various clinical data records to establish relationships. However, due to careful trial preparation and conscious efforts to follow SOPs for data collection and management, the data management team cleaned, corrected and properly joined data from various sources.

Despite all the trial preparation activities, the approach taken during the initial design of the data entry application did not take into account that major changes would need to be implemented in the CDMS during the course of the trial. The data management group attempted to reduce the effort necessary to implement new modules in the system, and these efforts, combined with the original design, formed the final architecture.

Given the extensive data entry validation requirements, the approach taken with low level of database normalisation and the database design was, initially, to leave validation-related code in separate application. This greatly simplified the database design and coding. Reporting was done with external system in SAS using data exported from database. This made data entry and database design less dependent from reporting. It could have been possible to follow all standard database design techniques instead of using straight forward copies of paper forms in database design; this design complicated implementing changes in database. This is a question about which tools the personnel is already familiar with and how much already existing code can be reused. For some institutions in the ARIVAC consortium SAS as a major statistics programming environment was a natural choice for many tasks in data validation and reporting. Problems arose in meeting deadlines for the verbatim report for the SAE prepared by the local safety monitoring team. Lack of coordination among the data managers and the manual computation of the figures to be included in the report were some of the causes for the delay.

The trial recruited local statisticians who learned how to manipulate data management and statistical software through regular trainings provided by the UQ data management experts. Although the CDMS evolved, the locally hired data management team became skilled in operating the system. There was considerable turnover in the data management team, but new staff quickly and efficiently gained knowledge of the system after training sessions conducted by the UQ.

Data collection officially ended December 2004. Some field trial staff, i.e. study nurses and physicians remained until end of 2005 to answer questions arising from the data processing. Data management team personnel, particularly the statisticians, were still available part time until 2009, when the main trial results were published [3]. An additional study of the geographic locations of children’s homes and services in the study area took place in years 2008–2009 with core members of the data management team [10]. Because of the skills that had been acquired, the local data management team was able to apply to this and other new research projects that had hired them. This was a great benefit to the research community at the RITM, in Manila. Continuation of data management is also important for the future use of the data for research and personnel familiar with the data solves queries of the researchers fast and reliably.

At the time of this trial, the data collection in the developing country settings had to rely on paper CRFs and recent development towards Electronic Data Capture (EDC) and/or web based distributed data collection and management was not considered [11]. At the time of the trial, the BHS had neither computer systems nor advanced telecommunications systems to support distributed data collection. Therefore, data collection and queries for quality control had to rely on paper forms, phone calls and fax [12].

We have hereby shown that in this complex, randomized, controlled trial to determine the efficacy of an investigational pneumococcal conjugate vaccine against childhood pneumonia (conducted in a developing country setting and involving concurrent satellite studies) data management needs/requirements were successfully met. There were several factors that made the task of data management doable and efficient. First, a pre-trial data management system established during the preparatory hospital based and immunogenicity studies in the population to obtain important background information about pneumonia, gave the trial a head start to conduct the data management tasks. Secondly, enough human resources like statisticians, programmers and support staff were recruited to help in data collection and management. Thirdly, the personnel had undergone training by experienced senior statisticians and programmers. Moreover, updating training was conducted locally and internationally throughout the study period. Fourthly, there was a health care data infrastructure utilizing the English language in the health records, which contributed to the ease of clinical data collection in this international project. Finally, a centralized data entry system combined with a semi-automated quality control resulted in a fairly fast feedback of corrections and high quality data with low error rates.

Although data management was successfully conducted, some problems arise during the trial. Trusting the functionality and reliability of the data management system building up the final database for data analyses, a full-time statistical expert from Australian collaborators was not hired for the entire duration of the trial to supervise the local data analyses [13]. We therefore encountered problems inherent to long-distance communication with the international statistical experts. Also the distributed administrative responsibilities of the consortium and unclear roles in new situations sometimes delayed decision making. Since the vaccine was an investigational product which was dropped from further licensure pathway, producer withdraw from the project and did not support post project data management or analysis. The need for human resources for post processing and analysis was underestimated. All this resulted in a one-year delay in providing the first results of the trial (Table 3).

**Table 3:** Summary table of key learnings

**Do’s**	**Don’ts**
To function effectively and efficiently, careful trial preparation and conscious efforts to follow SOPs for data collection and management should be done	Sometimes missing coordination of responsibilities and operational leadership in such distributed project
The international collaboration of data management personnel from the Philippines, Australia, and Finland managed to work effectively through different means of communication	The vaccine did not go further in licensure path – manufacturer withdraw and thus rest of consortium took response of management – reorganizational difficulties
Having committed and trained data management personnel is one of the significant factors in the conduct and success of a large clinical trial	Allocation of resources for post processing of data – timetable was stretched

## Conclusions

International research in developing country with many counterparts is challenging. To be successful one needs to deliver resources correctly to different places, to different tasks and on right time. It is important to find out what kind of resources, especially different professionals are available and where. This demand can be fulfilled by training new professionals, hiring existing ones or outsourcing services. Language and cultural barriers needs to be crossed to make such a large project working. In this case we did all these things keeping in mind sustainable capacity building in local institutions in the Philippines.

## Abbreviations

AOM, Acute otitis media; ARIVAC, Acute Respiratory Infection Vaccine Consortium; BCG, Bacillus Calmette Guérin vaccine; BHS, Barangay health station; BRH, Bohol Regional Hospital; CDMS, Clinical data management systems; CRF, Case report form; CSF, Cerebrospinal fluid; CU, University of Colorado, USA; DD, Data dictionary; DMG, Data manager; DSMB, Data safety monitoring board; DTP, Diphtheria, Tetanus, Pertussis combined vaccine; DTP/PRP ~ T, Diphtheria, Tetanus, Pertussis combined vaccine reconstituted with Haemophilus influenzae type b vaccine; EDC, Electronic data capture; EPI, Expanded program of immunization; HBV, Hepatitis B plasma derived vaccine; ICL, Imperial College London, UK; LAN, Local area network; LGU, Local government unit; OPD, Outpatient department; OPV, Oral poliomyelitis vaccine; PATH, Program for Appropriate Technology in Health; PCV, Pneumococcal conjugate vaccine; RCT, Randomized controlled trial; RDMS, Relational database management system; RITM, Research Institute for Tropical Medicine; SAE, Serious adverse event; SMS, Short message service; SOP, Standard operating procedure; THL, National Institute for Health and Welfare (Helsinki, Finland); UQ, University of Queensland; WHO, World health organization.

## Competing interests

The authors declare that they have no competing interests.

## Authors’ contributions

DHMS was the data manager of the project and wrote the initial draft of the manuscript. MGL, HN, VLT, and AT participated in drafting the manuscript, contributed ideas and provided comments to the manuscript. LTN participated in drafting the manuscript, and incorporated the revisions, comments and recommendations of the authors. GW was the biostatistician of the project who supervised the design and implementation of the data management systems, extensive quality control, as well the construction of the data warehouse for analysis and provided comments to the manuscript. All authors read and approved the final manuscript. ARIVAC Consortium consists of Joan Adamson; Adam Chester; Simon Forsyth; Elja Herva; Helena Mäkelä, MD; Antti Nissinen; Taneli Puumalainen, MD; Petri Ruutu, MD; Socorro Lupisan, MD; Beatriz Quiambao, MD; Ian Riley, MD and Eric Simões, MD.

ARIVAC Consortium consists of Joan Adamson; Adam Chester; Simon Forsyth; Elja Herva; Helena Mäkelä, MD; Antti Nissinen; Taneli Puumalainen, MD; Petri Ruutu, MD; Socorro Lupisan, MD; Beatriz Quiambao, MD; Ian Riley, MD and Eric Simões, MD
